# A Co-operative Regulation of Neuronal Excitability by UNC-7 Innexin and NCA/NALCN Leak Channel

**DOI:** 10.1186/1756-6606-4-16

**Published:** 2011-04-13

**Authors:** Magali Bouhours, Michelle D Po, Shangbang Gao, Wesley Hung, Hang Li, John Georgiou, John C Roder, Mei Zhen

**Affiliations:** 1 Samuel Lunenfeld Research Institute, Mount Sinai Hospital, Toronto, Canada M5G 1X5; 2 Department of Molecular Genetics, University of Toronto, Canada M5S 1A8

## Abstract

Gap junctions mediate the electrical coupling and intercellular communication between neighboring cells. Some gap junction proteins, namely connexins and pannexins in vertebrates, and innexins in invertebrates, may also function as hemichannels. A conserved NCA/Dmα1U/NALCN family cation leak channel regulates the excitability and activity of vertebrate and invertebrate neurons. In the present study, we describe a genetic and functional interaction between the innexin UNC-7 and the cation leak channel NCA in *Caenorhabditis elegans *neurons. While the loss of the neuronal NCA channel function leads to a reduced evoked postsynaptic current at neuromuscular junctions, a simultaneous loss of the UNC-7 function restores the evoked response. The expression of UNC-7 in neurons reverts the effect of the *unc-7 *mutation; moreover, the expression of UNC-7 mutant proteins that are predicted to be unable to form gap junctions also reverts this effect, suggesting that UNC-7 innexin regulates neuronal activity, in part, through gap junction-independent functions. We propose that, in addition to gap junction-mediated functions, UNC-7 innexin may also form hemichannels to regulate *C. elegans' *neuronal activity cooperatively with the NCA family leak channels.

## Introduction

Neuronal gap junction proteins mainly function at electrical synapses, where they form intercellular channels for ions and small molecules, such as second messengers, to flow bi-directionally, and sometimes directionally, allowing the coupled cells to synchronize their activities. In the nervous system, gap junction protein expression is tightly controlled throughout development [1-4]. Transient electrical coupling between neurons during early development has been proposed to affect the maturation and wiring of the adult nervous system [5,6]. In the mature brain, gap junction proteins, which are present in both neurons and glial cells [7], play critical roles in many physiological events [3,8] such as long range calcium wave propagation [9], neuronal plasticity [10] and REM sleep [11]. Gap junction proteins are also involved in pathological processes [12], in particular epilepsy [13] and hypoxia-ischemia response [14], where chemical or genetic inhibition of gap junctions was shown to help prevent the pathogenesis.

Vertebrate and invertebrate gap junctions exhibit similar structures and conserved functions. A gap junction is composed of two tightly associated, apposing hemichannel complexes between two neighbouring cells. A hemichannel is a hexamer complex composed of proteins with four transmembrane domains [15,16]. While invertebrate gap junctions are composed exclusively of innexins, vertebrates possess two classes of gap junction proteins: connexins and pannexins [17]. The role of connexins in mediating electrical cell coupling in the central nervous system (CNS) has been extensively documented [18-21]. Moreover, some connexins play roles in cell differentiation (reviewed in [22]), adhesion and migration (reviewed in [23]) that may be independent of their channel activities. These extensively studied connexins are topologically similar to innexins, but share little primary sequence homology, and have thus likely evolved independently from innexins [17,24]. On the contrary, pannexins were first identified through the sequence homology to innexins [25]. While Pannexin-3 is restricted to skin tissues, Pannexin-1 is expressed ubiquitously, but at high levels in the developing brain and Pannexin-2 appears restricted to the CNS. Pannexin-1 and Pannexin-2 are amongst the most abundant gap junction-forming proteins in the mammalian brain [17,25-28].

Some connexins participate in non-junctional forms of neuronal communication [29]. For example, connexins Cx46 and Cx50 were proposed to function as hemichannels to regulate the current homeostasis of lens [30]. Pannexin-1 can also form functional hemichannels under physiological voltages [28]. In pyramidal neuron postsynaptic termini, Pannexin-1 hemichannel was activated by NMDAR stimulation, which led to an epileptic seizure-like activity [31]. Oxygen/Glucose deprivation triggered the activation of Pannexin-1 hemichannel activity that contributed to the neuronal cell death [32]. Pannexins are also speculated to affect synaptic transmission, since they are present at postsynaptic regions of rodent hippocampal and cortical neurons [33]. However, the physiological role of pannexins, either at electrical synapses or in controlling neuronal activity, remains to be determined.

Gap junction hemichannels can be involved in another form of neuronal communication, referred to as ephapses [34,35]. This general term refers to a neuron's ability to influence neighbouring neurons' activity independently of electrical or chemical synapses. Ephapses may utilize several mechanisms [36,37], such as electric field effects and neuronal activity-related leakage of small molecules into the extracellular media [34]. In some tissues, such as the retina [36,37] and the olfactory nerve [38], ephaptic communication involves gap junction proteins, which function as hemichannels to mediate the leak of ATP into the extracellular and intersynaptic spaces, and to affect adjacent cells' activity through ATP-activated intracellular signalling (reviewed in [29] and [39]).

While 21 connexins and 3 pannexins are present in mammals [40], the *C. elegans *genome encodes 25 innexins, 19 of which are reported to be present in at least subsets of neurons [41]. Two of them, UNC-7, and its close homologue UNC-9, are expressed fairly broadly in many neurons, and more sporadically, in some muscle cells [42-45]. The loss of *unc-7 *innexin results in defects of the neuromuscular system, such as an inability of the animals to generate sinusoidal locomotion, hyperactivity of the egg-laying circuit [46], and strong resistance to aldicarb, a cholinesterase inhibitor [42]. Furthermore, *unc-7 *mutants exhibit synaptic disorganization along the nerve cord [42,47]. Despite obvious neuromuscular phenotypes, the underlying mechanism through which *unc-7 *mutations lead to these defects remains unclear. UNC-7 is expressed throughout development, with highest expression during the early larval stages [46]. Endogenous UNC-7 expression is detected in some sensory and many interneurons of the CNS, as well as in most motoneurons [43]. It is present both at presumptive gap junctions, and in at regions along the neurite that do not readily correlate to gap junctions defined anatomically by serial EM reconstruction ([42] and our unpublished observations). UNC-7 forms gap junctions with either itself, or UNC-9 when ectopically expressed in *Xenopus *oocytes, and between the AVB interneuron and B motoneurons *in vivo *[45]. UNC-7 also affects the development of presynaptic structures called active zones in GABAergic motoneurons, but functions in a cell-autonomous fashion, suggesting a gap junction-independent role [42].

*unc-7 *was reported to be genetically epistatic to *unc-79 *and *unc-80 *for volatile anesthetic response [48,49]. While *unc-79 *and *unc-80 *loss of function mutants are hypersensitive to halothane and chloroform, the loss of function mutations in UNC-7 innexin fully rescues this hypersensitivity. UNC-79 and UNC-80 were recently shown to be subunits of an evolutionarily conserved, novel cation channel complex called NCA in *C. elegans*, Dmα1U in *Drosophila *and NALCN/VgCN1 in vertebrates [50-54]. The NCA/NALCN channels regulate neuronal excitability and the propagation of neuronal activity [50-54]. The genetic interactions between *unc-79/unc-80 *and *unc-7 *therefore suggest a potential role for UNC-7 innexin in neuronal activity. Moreover, we noted that a hyper-activation of the NCA channel leads to several phenotypic characteristics similar to those exhibited by the *unc-7 *loss of function mutants, including the abnormal distribution of active zones in cholinergic and GABAergic motoneurons [42,54], and strong resistance to the pharmacological reagents aldicarb [42] and levamisole (data not shown) that perturb the activity of cholinergic neuromuscular junctions (NMJs). These lines of evidence further suggest a potential functional interaction between NCA and UNC-7. The observation that the expression pattern of endogenous UNC-7 innexin is broader than where gap junctions have been described [42] supports a possibility that its activity is not restricted to gap junction-related functions. We explored a possibility that in addition to gap junctions, the UNC-7 innexin may also function as 'leaky' hemichannels in neuronal membranes to modulate neuronal activity.

In the present study, through *in vivo *electrophysiology studies, we further identified an antagonistic genetic interaction between the NCA and UNC-7 channels in the context of NMJ activity. Moreover, we present evidence that UNC-7 innexin may function, at least in part, as a hemichannel to cooperatively regulate neuronal activity with the NCA channel.

## Materials and methods

### Strains

**Figure 1:**
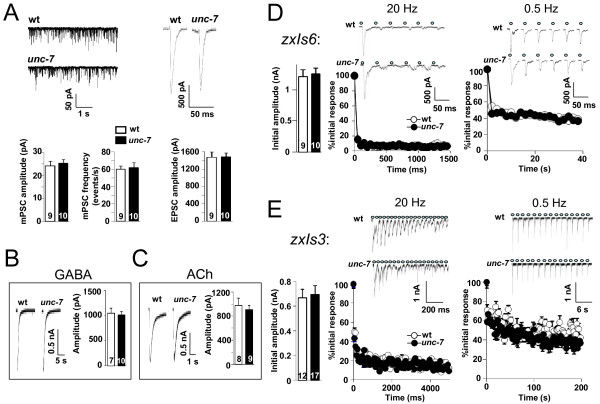
**A loss of UNC-7 function does not affect NMJ synaptic transmission**. (A) Representative traces of spontaneous mPSC (left) and electrically evoked EPSC (right) in wild type (wt) and *unc-7 *mutant animals (upper panels). Lower panels show the quantification of spontaneous mPSC amplitude and frequency, and electrically evoked EPSC amplitude in wild type (wt) and *unc-7 *animals. (B, C) GABA and acetylcholine receptors in *unc-7 *mutants were functional. Pressure ejection of GABA (0.1 mM) (B) or acetylcholine, Ach (0.1 mM) (C) evoked similar EPSCs in *unc-7 *mutant as in wild type animals. Detection of cholinergic (D) and GABAergic (E) light-evoked synaptic responses (upper traces) in animals expressing ChR2 in the corresponding motoneurons (*zxIs6 *and *zxIs3*, respectively). Quantifications of amplitude from light-evoked responses in wild type and *unc-7 *animals are shown in the lower panels. Animals were subjected to trains of stimulations at 20 Hz (left panels) and 0.5 Hz (right panels).

All strains were cultured at 22°C as described previously [42,47]. The Bristol N2 strain was considered and referred as the wild type in this study. CB5 *unc-7(e5) *was obtained from the *Caenorhabditis Genetics Center (CGC) *and outcrossed against N2 two times. ZM319 *unc-7(hp121) *was identified in a genetic screen for mutants defective for the GABAergic active zone marker *hpIs3 *(*Punc-25*-GFP::SYD-2), and outcrossed 4 times against N2 [42]. Both *unc-7 *alleles harbor early stop codons and are genetic and protein null alleles [42,45]. The two *unc-7 *alleles show similar behavioral and electrophysiological phenotypes (Figure 1 and data not shown). *nca-2(gk5) *and *nca-1(gk9) *were generated by the *C. elegans *Gene Knockout Consortium. *nca(lf) *(*nca-2(gk5);nca-1(gk9*)) were outcrossed over 7 times against N2. ZM1344 *hpIs61 *(*Punc-25*-GFP::UNC-10) was used for active zone morphology analysis [42]. ZX426 *zxIs3 *and ZX460 *zxIs6 *strains, expressing channelrhodopsin-2 (ChR2) in GABAergic and cholinergic neurons, respectively, were received from A. Gottschalk (U. Frankfurt, Germany).

### Constructs

pJH641 is a *C. elegans *expression vector that expresses *unc-7 *cDNA under the pan-neuronal promoter *PF25B3.3 *[55]. UNC-7 lacking extracellular cysteine residues (Cys-less) was constructed by serial mutations of the cysteine residues to alanines at amino acids 173, 191, 377 and 394 from pJH641. The mutated cDNA insert was sequenced and re-subcloned into a pan-neuronal promoter vector (pJH625) to give rise to pJH1300. Similarly, the single cysteine mutant of UNC-7 was generated in pJH641 to give UNC-7 C191A and re-subcloned in pJH625 to generate pJH1380. For neuronal subtype-specific promoter constructs, the *unc-7 *cDNA fragment from pJH641 was subcloned into the *Pglr-1 *(pJH528) and *Pacr-2 *(pJH1456) vector to generate interneuron- and cholinergic neuron-specific expression plasmids, pJH1556 and pJH1608, respectively.

### Transgenic *C. elegans *lines

Extrachromosomal arrays (*hpEx *lines) were generated by co-injecting the respective plasmids with a *Podr-1*-GFP injection marker. Strains carrying various transgenic arrays were used for the following experiments: 1) For electrophysiology and immunofluorescence studies: ZM3224 *nca(lf); unc-7(e5)*; *hpEx1171*[pJH641+*Podr-1*-GFP], ZM3306 *nca(lf); unc-7(e5); hpEx1218*[pJH1300+*Podr-1*-GFP], ZM3645 *nca(lf); unc-7(e5); hpEx1383*[pJH1380 +*Podr-1*-GFP]; 2) For aldicarb-response experiments, ZM1917 *unc-7(hp121); hpEx428*[pJH641+ *Podr-1*-GFP], ZM5215 *unc-7(hp121); hpEx2204*[pJH1300+*Podr-1*-GFP], ZM4376 *unc-7(hp121); hpEx1677*[pJH1380+*Podr-1*-GFP], ZM1912 *unc-7(hp121); hpEx433*[pJH1608 +*Podr-1*-GFP], ZM4705 *unc-7(e5); hpEx1577*[pJH1556+*Podr-1*-GFP]; 3) For active zone morphology analysis, ZM3164 *unc-7(e5); hpIs61; hpEx1135*[pJH641+*Podr-1*-GFP], ZM4949 *unc-7(e5); hpIs61; hpEx1996*[pJH1380+*Podr-1*-GFP], ZM4918 *unc-7(e5); hpIs61; hpEx1975*[pJH1300+*Podr-1*-GFP].

### Electrophysiology analyses in *C. elegans *and in Neuro2A cells

Dissections of young adult *C. elegans *were performed as described [56]. The integrity of the anterior ventral medial body muscle and the ventral nerve cord was visually examined, and muscle cells were then patched using fire-polished 4-MΩ resistant borosilicate pipettes (World Precision Instruments). They were clamped at -60 mV using a Multiclamp 700A amplifier (Molecular Devices) throughout experiments, and recorded by whole-cell patch-clamp using previously described recording solutions [54] within 10 min of the dissection. Signals were filtered at a lowpass frequency of 5 kHz and sampled at a rate of 50 kHz (interval 20 μs), and digitized via a Digidata 1440A converter (Molecular Devices). Data were acquired using pClamp10 software (Molecular Devices). After 10-60 s of recording spontaneous events, evoked responses were elicited. For electrically-evoked responses, a highly resistant fire-polished electrode filled with 3M KCl was brought close to the ventral nerve cord region at least 1 muscle cell length anterior to the recorded muscle cell, and a 1 ms depolarizing current, generated by a DS8000 Digital Stimulator (World Precision Instruments) was applied, its amplitude slightly adapted to induce a consistent, 1-2 nA evoked response in wild type animals. For photo-evoked responses, computer-controlled series of flashes of 450-490 nm blue light were applied to the whole preparation using a KSL70 LED controller (Rapp OptoElectronic, Germany) to stimulate the channelrhodopsin-expressing subsets of motoneurons as described [42]. For chemically-evoked responses, short pulses of ACh (0.1 mM) and GABA (0.1 mM) (Sigma-Aldrich) were applied to the recorded site of the muscle cell by a second electrode connected to a computer-driven Picospritzer III (Parker Instrumentation) pressure-ejection system. Data were analyzed with Clampfit10 (Axon Instruments) and graphed with Excel (Microsoft). Student's t-test was applied to analyze all electrophysiological data to determine statistical differences. In the figure plots, statistical differences at p < 0.05, p < 0.01, and p < 0.001 were considered statistically significant, and shown as single (*), double (**), or triple (***) asterisks, respectively.

Neuro2A cells were assessed for electrical coupling on the same setup. After the transfection (see below), cells were bathed in an extracellular solution containing (in mM): 140 NaCl, 10 glucose, 5 Hepes, 4 KCl, 2 CaCl_2_, 1 MgCl_2_, adjusted to pH7.4 with 1 mM NaOH and to 310 mOsm with sucrose. 3 MΩ-resistant recording electrodes were filled with the intracellular solution containing (in mM): 130 CsCl, 2 MgCl_2_, 10 Hepes, 4 Na_2_ATP, 10 glucose, adjusted to pH7.4 with KOH and to ~330 mOsm with sucrose. Adjacent cells both with bright GFP-fluorescence were clamped at the same voltage (-80 mV), and alternately applied 2s step voltages to +90 mV. Coupling was assessed by the occurrence of a slow, poorly voltage-dependent, non-inactivating current in one cell, in response to a step voltage applied to the other cell. Hemichannel currents were detected by step voltage stimulation from -120 to +100 mV in increment of 20 mV for 100 ms.

### Neuro2A cell culture, transfection and immunofluorescent staining

Neuro2A cells were cultured in 60 mm culture dishes for maintenance and electrophysiology analyses, and on glass coverslips for immunofluorescent staining. They were kept at 37°C, 5% CO_2 _and saturated humidity, and grown in MEM high glucose media supplemented with 10% Fetal Bovine Serum (Invitrogen), 1X Non-essential Amino Acids (Invitrogen), 1 mM sodium pyruvate (Invitrogen), 100U Penicillin, and 100 ug/mL Streptomycin (Invitrogen). When reaching 70-80% confluence, cells were dissociated with 0.25% trypsin (Invitrogen) in 1X PBS, and replated at 15% confluence for maintenance and 70% confluence for transfections. 4 μg of DNA per well was transfected into Neuro2A cells with Lipofectamine 2000 (Invitrogen) following the manufacturer's protocol.

Mammalian expression plasmids are pTracer-CMV (Invitrogen) based constructs. *unc-7 *and *unc-7 *cys-less cDNA were subcloned into a modified pTracer-CMV to give rise to pJH1874 and pJH1879, respectively. In both constructs, the CMV promoter drove the expression of UNC-7 (wild type or Cys-less form), while the EF1 promoter drove the expression of GFP for identification of transfected cells.

For immunofluorescent staining, 24 h, 48 h and 72 h after each transfection, Neuro2A cells were washed in PBS(+Mg^2+^/Ca^2+^), fixed with 4% PFA for 20 minutes, washed and kept in PBS at 4°C until staining. They were permeabilized in 0.2% Triton X-100 for 5 minutes, washed in PBS, followed by blocking with 1% Bovine Serum Albumin for 15 minutes. Cells were then incubated overnight with rabbit anti-UNC-7 antibody [54] at 1/300 in 1% BSA-PBS at room temperature. Secondary antibodies, Alexa Fluor 594 donkey anti-rabbit were used at 1/500 in BSA-PBS to stain the cells at room temperature for 30 minutes. Cells were washed with PBS and mounted with DABCO on glass slides for confocal imaging. Confocal images were obtained with a Nikon confocal microscope.

### *in vivo *imaging and quantification of the active zone number in *C. elegans*

Quantification of the fluorescent marker *hpIs61 *(*Punc-25-*GFP::UNC-10) in intact *C. elegans *was performed by visual examination and counting of fluorescent puncta along the entire dorsal nerve cord in L4 stage animals as previously described [42]. All scores were normalized against the wild-type animals scored on the same day, unless otherwise specified. Examiners were blinded to the genotype of strains examined, and these experiments were repeated more than four times.

### Aldicarb tests

Aldicarb plates were prepared as described previously [42]. At t = 0, ten 24 hr post L4 stage animals were put on each plate of a given aldicarb (Sigma) concentration, one plate per strain to be tested. The percentage of mobile animals after 240 minutes of aldicarb exposure at indicated concentrations was measured. Paralysis was defined as a straight posture and rigidity, combined with the lack of forward and backward movement when they were poked gently on the head twice. The same protocol was followed on the same day for each concentration of aldicarb. All strains shown on a graph were assayed on the same day, and the graphs are representative of a trend observed in at least three independent assays. Data were obtained by an examiner blinded for genotypes of strains being tested during the assay.

## Results

### Innexin *unc-7 *loss of function mutants do not display obvious synaptic transmission defects at NMJs

Defects associated with the *unc-7 *loss of function mutations, such as uncoordinated locomotion (Additional files 1 &2: Movies 1, 2), irregular active zone development and distribution along the nerve cords, and strong aldicarb resistance [42], all suggest a potential synaptic transmission deficit at NMJs. We addressed this possibility by recording the postsynaptic activity of the ventral body wall muscle at rest, and under various types of motoneuron stimulation *in situ *by voltage-clamp on dissected wild type and *unc-7 *null (*e5 *or *hp121*) animals. As both *e5 *and *hp121 *alleles harbor early stop codons, are protein-null alleles for UNC-7, and exhibit identical phenotypes [42,45], they were used interchangeably as *unc-7 *null mutants in this study.

To our surprise, we were unable to detect any obvious alteration of NMJ properties in *unc-7 *mutants under our experimental conditions. In the absence of stimulation, the amplitude and frequency of the body wall muscle miniature postsynaptic currents (mPSCs) were identical between wild type and *unc-7 *animals. The evoked postsynaptic currents (EPSCs) in response to electrical stimulation of the motoneurons (electric-EPSCs) were also comparable in kinetics and amplitude between wild type animals and *unc-7 *mutants (Figure 1A, Additional file 3: Table S1).

We further tested the possibility that synaptic scaling or other postsynaptic compensatory mechanisms may have accounted for the lack of synaptic transmission phenotypes. *C. elegans' *NMJs utilize either Acetylcholine (ACh) or Gamma-Aminobutyric-Acid (GABA) to induce muscle contraction or relaxation, respectively [56-58]. By pressure-ejecting respective neurotransmitters at body wall muscles and recording subsequent EPSCs, we tested the sensitivity of the postsynaptic membrane to ACh and GABA. Both neurotransmitters evoked EPSCs with similar amplitude and kinetics in wild type and *unc-7 *animals (Figure 1B and 1C, Additional file 3: Table S1), indicating the lack of significant compensation at postsynaptic membranes.

We wondered whether UNC-7 may be more important during high levels of synaptic activity. Electrical stimulation of the nerve cord results in mostly cholinergic response, excluding the analyses of GABAergic synapses [56]. Furthermore, because it rapidly damages the preparation, it is less ideal for the study of tetanic modulation [56,59]. To address the possibility that UNC-7 may affect some forms of synaptic plasticity at NMJs, we instead used transgenic lines expressing channelrhodopsin-2 (ChR2) in either cholinergic or GABAergic neurons, and recorded EPSCs activated by either type of motoneurons upon trains of blue light stimulation (photo-EPSCs) [56]. Photo-EPSCs by cholinergic motoneurons were indistinguishable between wild type and *unc-7 *animals (Figure 1D). The amplitude of the initial response was identical (Figure 1D, Additional file 3: Table S1), and the decay of photo-EPSCs amplitudes during 0.5 or 20Hz trains of stimulations shared the same kinetics, plateaued similarly at ~10% of the initial response amplitude at 20Hz, and ~40% at 0.5Hz (Figure 1D). Photo-EPSCs by GABAergic motoneurons were also similar between wild type and *unc-7 *mutant animals in amplitude and kinetics (Figure 1E, Additional file 3: Table S1).

**Figure 2:**
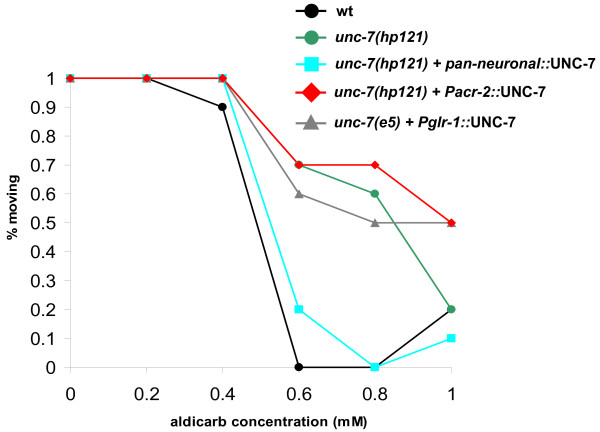
**Tissue specificity of UNC-7 expression for aldicarb phenotype**. *unc-7(hp121) *animals expressing UNC-7 by pan-neuronal (*Prgef-1*), cholinergic (*Pacr-2*) and interneuron (*Pglr-1*) promoters were assayed for aldicarb sensitivity. Representative results of a same-day experiment on the percentage of mobile animals after 240 minutes of aldicarb exposure at indicated concentrations (see Methods, n = 10 animals per strain). Pan-neuronally expressed UNC-7 rescued aldicarb sensitivity, but expression of UNC-7 in cholinergic or interneurons failed to rescue aldicarb sensitivity.

Taken together, these results indicate that the locomotion phenotype, pharmacological response, as well as synaptic morphological defects of *unc-7 *mutants are unlikely to result from a simple deficit in synaptic transmission at NMJs. Indeed, the strong resistance to aldicarb, a cholinesterase inhibitor that perturbs cholinergic synaptic transmission, exhibited by *unc-7 *mutants, could only be consistently restored by pan-neuronal expression of UNC-7 ([42]; Figure 2). The altered pharmacological response of *unc-7 *mutants may result from multiple neuronal types, or a cell type not tested.

### UNC-7 and NCA channels exhibit antagonistic, functional interactions at NMJs

**Figure 3:**
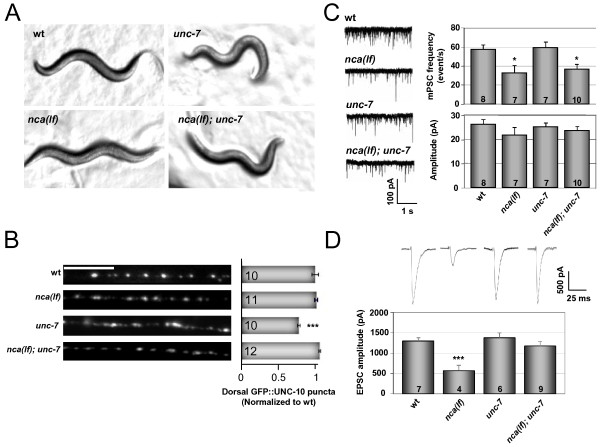
**UNC-7 and NCA functionally interact**. (A) A snap shot of usual postures on plates of wild type (smooth sinusoidal), *unc-7 *(irregular body bends and kinks)*, nca(lf) *(fainter and relaxed) and *nca(lf); unc-7 *(irregular body bends and kinks) animals. (B) *nca(lf) *rescued the *unc-7 *active zone defect. In wild type and *nca(lf) *animals, active zone marker GFP::UNC-10 (*hpIs61*) appeared round and evenly spaced. *unc-7 *animals showed more diffuse GFP::UNC-10 puncta, whereas GFP::UNC-10 puncta in *nca(lf); unc-7 *showed more evenly spaced and round morphology. Quantification of GFP::UNC-10 dorsal cord puncta (right panel) showed a significant decrease in the punctum number in *unc-7 *mutants when compared to wild type animals. Scale bar: 5 μm. (C) Representative traces of spontaneous mPSCs in animals with different genetic backgrounds (left panels). Quantification of mPSC frequency and amplitude is shown in the right panels. While mPSC frequency was reduced in both *nca(lf) *and *nca(lf); unc-7 *animals when compared to wild type animals, there was no significant difference in the mPSC amplitude by genotype. (D) *nca(lf); unc-7 *EPSCs were rescued back to *unc-7 *or wild type amplitude. Representative traces of evoked response from animals of different genetic background are shown. Quantification of EPSC amplitude was shown in the lower panel. *nca(lf) *showed significantly reduced EPSC amplitude when compared to wild type, while *unc-7 *mutation restored EPSC amplitude of *nca(lf) *to the wild type level. Student's t-test, *, p < 0.05; ***, p < 0.001.

To determine the role of UNC-7 at the neuromuscular system, we further examined its possible functional interactions with the NCA cation channel, first alluded to by their reported genetic interactions in volatile anaesthetic sensitivities [49,60]. In *C. elegans*, loss of function mutations in the functionally redundant pore-forming subunits (both NCA-1 and NCA-2, *nca(lf)*), or the auxiliary subunits of the channel complex, UNC-79 or UNC-80, result in a 'fainter' phenotype, where mutant animals are capable of normal locomotion when stimulated, but fail to sustain the activity and succumb to muscle relaxation shortly after [54]. Consistently, synaptic transmission at NMJs is reduced, both at rest and following the electric stimulation of motoneurons [54]. We generated and examined *nca(lf); unc-7 *triple loss of function mutants. They no longer faint but exhibit strong, sustained jerky locomotion characteristic of *unc-7 *mutants (Figure 3A, Additional file 2, 4, &5: Movies 2, 3 and 4). This suggests that *unc-7 *is also genetically epistatic to *nca(lf) *in regards to locomotory behavior.

Do NCA and UNC-7 also exhibit genetic interactions for the NMJ morphology? We analyzed the active zone morphology and distribution at GABAergic NMJs using the *hpIs61 *(*Punc-25*-GFP::UNC-10) marker; Figure 3B). The expression pattern of *hpIs61 *is normal in *nca(lf) *mutants, with bright, even GFP::UNC-10 fluorescent puncta regularly spaced along the dorsal nerve cord (Figure 3B), whereas *unc-7 *mutants, as previously reported, exhibit puncta with uneven brightness, size and distribution that correlated with the altered morphology and distribution of active zones by EM analyses ([42] and Figure 3B). The *unc-7 *mutants show a net decrease of the total bright GFP::UNC-10 puncta number (73.2 ± 1.8% of wild type, p < 0.001). In *nca(lf); unc-7 *animals, the active zone puncta appeared even in brightness, size and distribution, reflected by an increased total number of bright puncta that was similar to that of wild type (p > 0.05) or *nca(lf) *(p > 0.05) animals (Figure 3B). Therefore, for NMJ morphology, mutations in the NCA channel appear genetically epistatic to the loss of UNC-7, with *nca(lf) *rescuing the NMJ morphology defect of *unc-7 *mutants.

To assess whether the genetic interaction between *unc-7 *and *nca(lf) *was also reflected at a functional level, we examined the spontaneous and electrically evoked synaptic activity at the NMJs of *nca(lf); unc-7 *animals (Figure 3C and 3D, Additional file 3: Table S2). The frequency of mPSCs in *nca(lf); unc-7 *mutants (36.2 ± 5.5Hz, n = 10) was similar to that of *nca(lf) *animals (32.4 ± 8.3Hz, n = 7, p > 0.05). The mPSC frequencies for both *nca(lf); unc-7 *and *nca(lf) *were significantly decreased when compared to both wild type animals (57.3 ± 4.9Hz, n = 8, p < 0.05 and p < 0.05 respectively), and to *unc-7 *(59.0 ± 6.2Hz, n = 7, p < 0.05 and p < 0.05, respectively) (Figure 3C). The average mPSC amplitude was unaffected in any mutant (23.4 ± 2pA for *nca(lf); unc-7*, n = 10; 21.7 ± 3.4pA for *nca(lf)*, n = 7; 25.1 ± 1.9 pA for *unc-7*, n = 7) as compared to wild type (26.1 ± 2.2pA, n = 8, p > 0.05) (Figure 3C). The similarity of mPSCs characteristics between the *nca(lf) *and *nca(lf); unc-7 *mutants thus suggests a functional dominance by the NCA channel at NMJs at rest.

When the nerve cord was stimulated, the electric-EPSC amplitude of *unc-7 *animals (1.37 ± 0.13nA, n = 6) was identical to that of wild type animals (1.30 ± 0.08nA, n = 7, p > 0.05). While the response was reduced by approximately 60% (0.58 ± 0.13nA, n = 4, p < 0.001) in *nca(lf) *mutants ([54] and Figure 3D), the electric-EPSC amplitude of *nca(lf); unc-7 *animals (1.20 ± 0.10nA, n = 9) was identical to that of wild type (p > 0.05) and *unc-7 *mutants (p > 0.05). Therefore, upon stimulation, the effect of the UNC-7 channel dominates that of NCA, which is opposite to the situation at rest.

Overall, the genetic interactions between *nca(lf) *and *unc-7 *mutants are complex, as their epistatic relationships are phenotype-dependent. These interactions do, however, confirm the antagonistic interactions between these two channels, and further indicate a cooperative modulation of the activity and development of the neuromuscular system.

### The NCA/UNC-7 interaction depends strictly on neuronal UNC-7

The NCA channel is expressed and required in the nervous system. UNC-7, on the other hand, is expressed not only in neurons, but also in body wall muscles (albeit sporadically). Studies to date indicate that UNC-7 is either inactive or functionally redundant in muscles: the electrical coupling between body wall muscle cells is not affected by *unc-7 *mutations [44], and the locomotory defect of *unc-7 *mutants is of neuronal, but not of muscular origin [42,45]. Pan-neuronal expression of UNC-7 also fully rescued aldicarb resistance in *unc-7 *mutants and the active zone defects, while the expression of UNC-7 in muscles rescued neither phenotypes [42].

To test if UNC-7 functions in neurons to modify the NMJ function in the *nca(lf) *mutant background, we compared the effect of restoring UNC-7 expression in either neurons, or muscles in *nca(lf); unc-7 *mutants. The pan-neuronal expression of UNC-7 in *nca(lf); unc-7 *mutants led to a complete behavioral reversion to the fainter phenotype, identical to that of *nca(lf) *mutants (Additional file 4, 5, &6: Movies 3, 4, & 5). Accordingly, the EPSC amplitude of *nca(lf); unc-7 *mutants carrying a transgenic array expressing a pan-neuronal promoter-driven UNC-7 was also reduced by about 65% when compared to *nca(lf); unc-7 *animals not expressing the transgene (Additional file 3: Table S2). The decrease in EPSC amplitude was comparable to that observed between wild type and *nca(lf) *mutants, indicating that neuronal UNC-7 mediates such an interaction with NCA. These results further indicate that neuronal UNC-7 channel functions together with the NCA channel to modulate neuronal activities.

### Cys-less UNC-7 Innexin does not show gap junction-like localization

Pannexins are now proposed to function mainly as hemichannels, rather than gap junctions [61]. To examine whether UNC-7 affects neuronal activity in a gap junction-dependent or independent manner, we tested several mutant forms of UNC-7 that are predicted to prevent gap junction formation without significantly affecting hemichannel activity. In connexins, cysteines (Cys) in the extracellular loops form disulfide bonds that help maintain an extracellular β-sheet structure, and such an extracellular configuration provides the docking site for hemichannel interactions [62-64]. Substituting one or all of these Cys in Cx32 and Cx43 abolished their ability to form functional gap junctions [64,65] with a minimal effect on the hemichannel activity [66].

**Figure 4:**
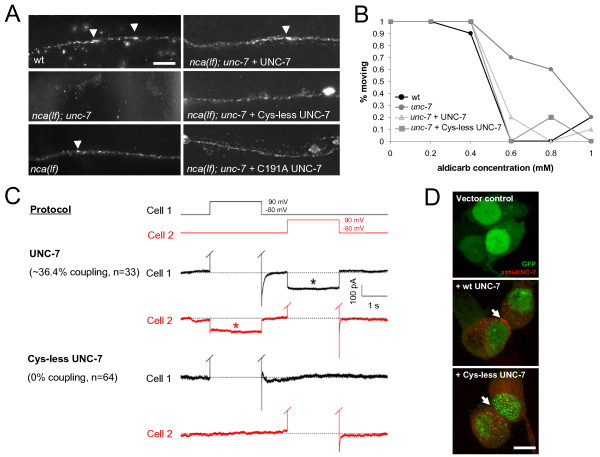
**Cys-less UNC-7 fails to localize to large junctional-like structures, but remains functional to rescue *unc-7 *aldicarb resistance**. (A) Ventral nerve cord images of anti-UNC-7 immunostaining in wild type, *nca(lf), nca(lf); unc-7 *and *nca(lf); unc-7 *animals expressing wild type UNC-7 and mutant forms of UNC-7 under a pan-neuronal promoter (*Prgef-1*). Endogenous UNC-7 in wild type and *nca(lf)*, as well as exogenously and pan-neuronally expressed UNC-7 in *nca(lf); unc-7 *all showed punctate distribution: bright and elongated patches resembling large junctional structures (arrowheads) and numerous dense punctate of unknown structures. Cys-less, or C191A UNC-7 eliminated these junctional-like patches, but left the smaller-sized densely populated punctate signal unchanged. Scale bar: 5 μm. (B) Representative results of same day experiments for aldicarb sensitivity after 240 min of exposure to aldicarb (n = 10). *unc-7(hp121) *mutants had increased aldicarb resistance when compared to wild type animals. Expression of either UNC-7 or Cys-less UNC-7 restored aldicarb sensitivity of *unc-7 *mutants to that of the wild type level. (C) Heterologous expression of wild type UNC-7 in Neuro2A cells allowed electric coupling between cells, whereas cells expressing Cys-less UNC-7 did not induce coupling. (D) Localization of exogenously expressed UNC-7 in Neuro2A cells. Transfected Neuro2A cells were stained with anti-UNC-7 antibody (red) and GFP expressed from the same vector in the transfected cells (green). Wild type UNC-7 localized at junctions of two adjacent cells (white arrow), whereas Cys-less UNC-7 did not. Scale bar: 10 μm.

Though innexins lack overall sequence similarity to connexins, they have a similar protein topology. Among innexins there are four extracellular Cys at conserved positions that are predicted to play an analogous role in gap junction docking as the extracellular Cys in connexins [67]. We expressed wild type or mutant forms of the UNC-7 protein, where the second (C191) or all four extracellular Cys were substituted by alanine, using a pan-neuronal promoter in *unc-7 *or *nca(lf); unc-7 *mutants. Their subcellular localizations were examined subsequently by immunofluorescent staining with UNC-7 antibodies. Strains expressing the pan-neuronal wild type UNC-7 displayed prominent staining along both ventral and dorsal nerve cord neurites, with both large patches (on average ~0.71 ± 0.38 μm in length) that likely correspond to gap junctions described by ultrastructural reconstruction of the *C. elegans *nervous system, and numerous, tiny puncta of unknown correlating structures (Figure 4A), reminiscent of the endogenous UNC-7 expression pattern [42,45]. Comparatively, the Cys-less UNC-7 staining was mostly retained in the neuronal cell bodies and was less prominent along the proximal, ventral processes (Figure 4A), and almost disappeared along the distal, dorsal nerve cord (not shown), suggesting that these Cys are important for effective trafficking of UNC-7 along neurites. Strikingly, along the ventral nerve cord, while all large UNC-7 fluorescent patches completely disappeared, many small puncta, although reduced, are still present (Figure 4A). This result is thus consistent with that Cys-less UNC-7, in addition to showing reduced intracellular trafficking, is also being defective in clustering and in the subsequent formation of gap junctions along the plasma membrane.

*unc-7 *mutants exhibited robust resistance to the cholinesterase inhibitor aldicarb. The pan-neuronal expression of C191A UNC-7 or Cys-less UNC-7 in *unc-7 *restored their aldicarb sensitivity to the same extent as the wild type UNC-7 (Figure 4B). UNC-7, therefore, likely affects the pharmacological response, as well as synaptic function, in a gap junction-independent manner. We then directly assessed the ability of exogenously expressed Cys-less UNC-7 to form gap junctions in Neuro2A cells. After transfection with plasmids that co-expressed either wild type or Cys-less UNC-7 with GFP, adjacent GFP-positive cells were recorded by dual-patch-clamping. 36.4% (12 out of 33) of the UNC-7-transfected cell pairs displayed obvious bi-directional electrical coupling, with the voltage change induced in one cell detected in its un-stimulated neighbour (Figure 4C). Such a trans-junctional current was never detected in untransfected cell pairs (n = 14), cells transfected with the empty vector (n = 23), and importantly, cells transfected with Cys-less UNC-7 (n = 64). Consistently, dense junctional UNC-7 staining along the contacting surface of the two neighbouring cells, which likely represented gap junctions, as well as intracellular UNC-7 staining was observed in Neuro2A cells transfected with wild type UNC-7 (Figure 4D). Cells transfected with Cys-less UNC-7, on the other hand, exhibited strong intracellular clusters, and scattered plasma membrane staining pattern not enriched along the aligned surface between the contacting cells (Figure 4D), confirming that UNC-7 Cys-less proteins are compromised in their ability to form or maintain gap junctions.

### Cys-less UNC-7 retains certain physiological roles in *C. elegans *neurons

We next examined whether these gap junction-defective UNC-7 proteins compromised UNC-7's physiological functions *in vivo*. Unlike wild type UNC-7, which fully restored *unc-7 *locomotion, expressing C191A UNC-7 or Cys-less UNC-7 by the same pan-neuronal promoter did not rescue locomotion defects (Additional file 7, 8, &9: Movies 6, 7, & 8). Similarly, the pan-neuronal expression of gap junction-defective UNC-7 proteins failed to revert the movement of *nca(lf); unc-7 *mutants to the characteristic *nca(lf)*-like 'fainter' locomotion (Additional file 10 &11: Movies 9 and 10). Therefore UNC-7 affects locomotion through gap junction dependent function.

**Figure 5:**
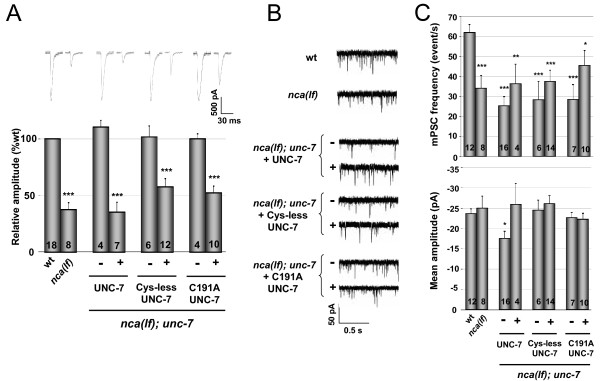
**Gap junction-incompetent UNC-7 maintains its functional interaction with NCA**. (A) Representative evoked synaptic responses (upper traces) and quantification (lower plot) of animals of various genotypes relative to that of wild type (wt) animals; significance tests were carried out in comparison to that of wt. Currents were recorded from animals expressing wild-type and mutant-form of UNC-7 constructs (+) in the *nca(lf); unc-7 *triple mutants, and their non-transgenic sibling from the same parents (-) were examined as controls. UNC-7 expression fully reverted *nca(lf); unc-7 *evoked responses to the *nca(lf) *level. Expression of Cys-less or C191A UNC-7 also reverted the evoked response. (B) Representative traces of spontaneous mPSCs from wild type (wt), *nca(lf) *and *nca(lf); unc-7 *animals, expressing various UNC-7 constructs by the pan-neuronal promoter. (C) Quantification of the frequency (upper plot) and amplitude (lower plot) of mPSCs from animals of various genetic backgrounds shown in (B). Transgenic animals expressing various UNC-7 constructs (+) and their non-transgenic siblings from the same parent as controls (-), were quantified by Student's t-test. * p < 0.05, ** p < 0.01, *** p < 0.001.

**Figure 6:**
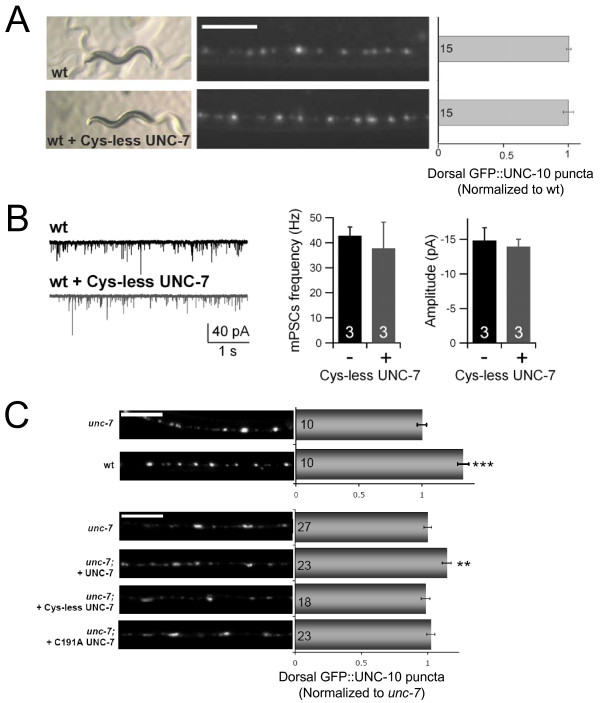
**An over-expression of Cys-less UNC-7 does not cause dominant negative effect**. (A) An over-expression of Cys-less UNC-7 mutant proteins did not have effect on active zone morphology. Wild type animals carrying the GFP::UNC-10 active zone marker were injected with a plasmid expressing Cys-less UNC-7 proteins. Morphology (left panels) and total number (right panel) of dorsal GFP::UNC-10 puncta were comparable in non-transgenic (wt) or transgenic (wt + Cys-less UNC-7) animals. Scale bar: 5 μm. (B) An over-expression of the Cys-less UNC-7 mutant proteins in wild type animals did not change the mPSC frequency or amplitude. Representative traces of mPSC recording from wild type (wt) animals or animals expressing Cys-less UNC-7 (wt + Cys-less UNC-7) were shown (left panel), and mPSC amplitude and frequency quantified (right panel). (C) An over-expression of wild type, Cys-less and C191A UNC-7 proteins in *unc-7 *mutant background. Wild type UNC-7 protein, but not the two mutant UNC-7 proteins, rescued active zone morphology defect. Total number of dorsal GFP::UNC-10 puncta were quantified and normalized to that of *unc-7*. Scale bar: 5 μm.

However, Cys-less UNC-7 proteins retain clear physiological functions in other assays. They fully rescued some NMJ phenotypes exhibited by *unc-7 *and *nca(lf); unc-7 *mutants. Consistently, the genetic and functional interactions between the NCA and UNC-7 channels are also partially independent on UNC-7's ability to form gap junctions. Although expressing pan-neuronal C191A or Cys-less UNC-7 in *nca(lf); unc-7 *mutants did not alter their locomotion, it did revert the rescuing effect of the *unc-7 *mutation on the evoked postsynaptic currents in body wall muscles: the electric-EPSCs in these transgenic animals ('+') were reduced by >40%, when compared to their respective controls (their non-transgenic siblings '-') (Figure 5A, Additional file 3: Table S2). Expressing wild type UNC-7 protein under the same neuronal promoter in *nca(lf); unc-7 *mutants led to a statistically similar degree of decrease in the EPSC amplitude, though the decrease mediated by wild type UNC-7 showed a tendency to be slightly larger than that by C191A or Cys-less UNC-7 (Figure 5A). Consistent with a loss of *unc-7 *not affecting NMJ properties at rest, expressing wild type or Cys-less UNC-7 in *nca(lf)*, *unc-7 *or *nca(lf); unc-7 *did not alter mPSC frequency or amplitude (Figure 5B and 5C, Additional file 3: Table S2 and data not shown). Moreover, Cys-less UNC-7 did not exert a dominant negative effect on behavior, NMJ morphology or functional integrity when over-expressed in wild-type animals (Figure 6A and 6B). These results imply that Cys-less UNC-7 proteins did not interfere, at least not at a physiologically significant level, with the trafficking and function of the wild-type UNC-7 protein. Taken together, these observations suggest that UNC-7's effect on neuronal activity also involves a gap junction-independent role.

Restoring wild-type UNC-7 panneurally or in GABAergic neurons alone in *unc-7 *mutants fully rescued the defective active zone distribution in DD motoneurons, suggesting a gap junction-independent function ([42], Figure 6C, and data not shown). A pan-neural expression of Cys-less UNC-7, however, led to variable results and did not result in a consistent rescue of the active zone defects (Figure 6C). Because we scored for the active zone in the distal dorsal process, where both large and small Cys-less UNC-7 clusters failed to be delivered due to the trafficking defects (data not shown), it is difficult to assess whether Cys-less UNC-7 is functional in regulating active zone morphology.

### Potential hemichannel activity of UNC-7 innexins in transfected Neuro2A cells

**Figure 7:**
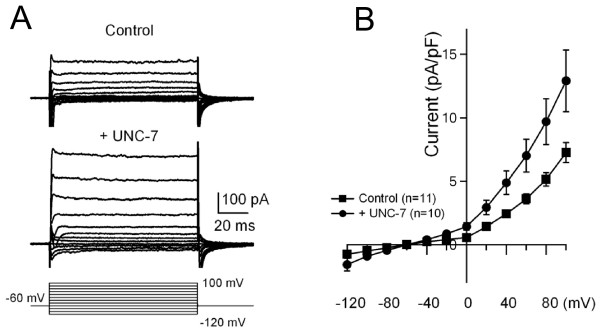
**UNC-7 exhibited hemichannel properties in Neuro2A cells**. (A) Representative current traces recorded in UNC-7 non-transfected (n = 11) and transfected (n = 10) Neuro2A cells, respectively. Currents were evoked by step voltage from -120 to +100 mV in increment of 20 mV. Cells were held at -60 mV. (B) Graphic presentation of the average current intensities. Significantly increased currents were observed in UNC-7 transfected Neuro2A cells.

We further examined if UNC-7 innexin could exert hemichannel activity when transfected in Neuro2A cells. Indeed, some unpaired UNC-7 transfected Neuro2A cells exhibited current activities upon voltage stimulation (Figure 7A), and the averaged current intensity was significantly increased in UNC-7 transfected Neuro2A cells versus cells transfected with empty vectors (Figure 7B). The same size Neuro2A cells were selected for recording (Control: 26.4 ± 1.9pF, n = 11; +UNC-7: 25.2 ± 1.9pF, n = 10, p > 0.05). Taken together, this result suggests that UNC-7 innexin may exert hemichannel activity when transfected in Neuro2A cells.

## Discussion

In the present study, we showed that, although *unc-7 *loss of function mutations do not lead to obvious synaptic transmission defects at NMJs, they partially rescued the reduced synaptic transmission in *nca(lf) *mutants, suggesting an antagonistic interaction between the NCA cation leak channel and the UNC-7 innexin channel in neuronal activity modulation. We further demonstrate *in vivo *and *in vitro *that neuronal UNC-7 exerts these functions in part through non-junctional activities. UNC-7 may function as both gap junctions and hemichannels to regulate different aspects of nervous system development and function.

### Disparity between behavior, aldicarb sensitivity and synaptic transmission of *unc-7 *mutants

While there have been reports of several *C. elegans *mutants that display superficially normal locomotion, but altered synaptic transmission at NMJs, the opposite example, mutants with strongly uncoordinated locomotion, of a neuronal origin and with altered synaptic morphology, but no detectable synaptic transmission defects at NMJs, such as the case for *unc-7*, are rare. Moreover, although *nca(lf); unc-7 *animals expressing Cys-less UNC-7 did not affect their locomotion, or they did show a full reversion of *unc-7*'s modifying effect on synaptic transmission at NMJs.

Why do mutations in UNC-7 result in locomotory and pharmacological defects that are often associated with altered cholinergic synaptic transmission, but have no detectable synaptic transmission alteration by electrophysiology analyses? One possibility is that UNC-7 does affect the motoneuron activity, but the effect was obscured when applying strong stimulations to these neurons as required for the current electrophysiological analyses. Another possibility is that the locomotory defects of *unc-7 *mutants result mainly from defects in neurons that send inputs to motoneurons, either in the activation and/or a coordination of motoneurons, which we would also bypass or mask through the direct motoneuron stimulation in the current electrophysiological analyses. It has indeed been shown that the major locomotory defects associated with *unc-7 *mutants could be rescued by restoring UNC-7 expression in a small set of command interneurons [45]. Given its broad expression in the nervous system, UNC-7 likely plays distinct roles, both developmental and functional, in different subclasses of neurons, to account for different aspects of phenotypes detected in *unc-7 *mutants.

### UNC-7 functions, in part, independently of gap junctions in *C. elegans *neurons

The strong rescuing ability of Cys-less UNC-7 proteins in the altered pharmacological response of *unc-7 *mutants and synaptic activity of *unc-7 *and *nca(lf); unc-7 *mutants implicated that, in addition to its role in electrical coupling, UNC-7 is also involved in roles independent of gap junctions to affect activity at NMJs. Below we propose two potential mechanisms through which non-junctional UNC-7 may affect neuronal activity. Both were based on studies of Pannexins, the closest mammalian homologues of innexins, which were proposed to primarily function independently of gap junctions [61].

Mouse taste receptor cells released ATP through Pannexin-1 hemichannels, which further triggered serotonin release from adjacent taste receptor cells [68,69]. Pannexin hemichannels were also shown to propagate ATP and calcium waves in response to ischemia-induced stimulation, or the liberation of nitric oxide, spreading the effect of ischemia during strokes [32,70,71]. By analogy, UNC-7 hemichannels may modulate the propagation of excitation signals along neurites, by modulating the intracellular level of ATP, calcium or other ions across the plasma membrane.

Another intriguing possibility is suggested by the study where pannexins were shown to play a role in calcium signalling and homeostasis at the ER [72]. In heterologous systems, Pannexin-1 appeared not only localized at the plasma membrane, but also intracellularly, where it regulated calcium release from intracellular calcium stores. Therefore UNC-7 may also act as an intracellular channel influencing intracellular calcium concentration under specific physiological conditions. Our co-immunostaining analyses with UNC-7 and an ER marker did not reveal convincing co-localization in *C. elegans *neurons (data not shown), but the resolution limit of *C. elegans *neurites prevented ruling out this possibility completely.

### UNC-7 functionally interacts with the NCA complex to regulate neuronal activity

The fact that the *unc-7 *mutants on their own did not exhibit obvious synaptic transmission defects at NMJs indicates either a functional redundancy between UNC-7 and other innexins, or that UNC-7 functions with/through other channels to modulate neuronal activity in either the same or different sets of neurons. Its antagonistic genetic interaction with the NCA channel strongly supports the latter possibility.

The NCA channel family is involved in setting the activation threshold, and therefore, the activity of neurons. In *C. elegans*, the NCA complex regulates NMJ activities and affects the propagation of activity-related calcium signals along neurites [50,54]. Its mammalian homologue NALCN is a poorly selective, voltage-independent cation leak channel that modulates the resting membrane potential and activity of hippocampal neurons [52]. NALCN was recently shown to mediate neurotensin- and substance P-induced membrane depolarization in the mouse hippocampus and ventral tegmental neurons [51].

The genetic interactions between the *nca(lf) *and *unc-7 *innexin mutants are of an antagonistic nature, suggesting that UNC-7 innexin regulates neuronal activity cooperatively with the NCA leak channel. Pannexins are proposed to either directly or indirectly modulate intracellular calcium. It is widely accepted that they form large, poorly selective channels in the plasma as well as intracellular membranes [73-75]. Together with results from our present study, it is conceivable that both the UNC-7 hemichannel and NCA channel function as leak channels to modulate neuronal membrane properties, but in an opposite fashion, and their activities are influenced by membrane properties established by each other. Alternatively, NCA may modulate the activity of an intracellular UNC-7 hemichannel to affect the intracellular calcium level and propagation.

The complex, antagonistic genetic interactions between *nca(lf) *and *unc-7*, with the *nca(lf) *phenotype being dominant at rest, and *unc-7 *being dominant when neurons were stimulated, may be explained by these channels being activated under different membrane/intracellular conditions. For example, it is possible that at rest, the UNC-7 hemichannel is closed, and this contributes to a membrane/intracellular property that potentiate the opening of the NCA channel to modulate the basal neuronal activity. *nca(lf); unc-7 *therefore resembles the phenotype of *nca(lf) *at rest. Upon strong stimulation, however, UNC-7 hemichannels are also activated, and NCA-dependent neuronal activity, such as the increase of intracellular calcium concentration or membrane potential, may be required to inhibit the UNC-7 hemichannel activity to prevent a further increase or prolongation of neuronal activity. Deleting UNC-7 in *nca(lf) *would therefore lead to an increase of evoked currents.

As previously reported, both NCA and UNC-7 are expressed in punctate patterns along neurites, and both appear excluded from synapses. However, our whole-mount co-immunofluorescent staining with antibodies against UNC-7 and NCA-1 did not show obvious co-localization (data not shown). Though not definitively conclusive due to the limited resolution afforded by the neurite, this further implies that the nature of the NCA and UNC-7 genetic interaction probably reflects a functional, rather than a physical interaction. A better understanding of the properties of the NCA leak and UNC-7 hemichannels, and a detailed examination of their functional requirements in specific neurons for different phenotypes, should help address the mechanistic details of such a functional relationship.

## Competing interests

The authors declare that they have no competing interests.

## Authors' contributions

MB and MZ designed the study and drafted the manuscript. MB and SBG performed electrophysiology experiments; MP and HL performed molecular biology experiments; MB performed all other experiments; WH, JG and JR offered advice and help with imaging and electrophysiology setup; MP, WH and JG edited the manuscript. All authors read and approved the final manuscript.

## Supplementary Material

Additional file 1 **Supplemental Movie 1**. wild type (N2) animal Click here for file

Additional file 2 **Supplemental Movie 2**. *unc-7 (e5) *animal Click here for file

Additional file 3 **Supplemental tables**. Click here for file

Additional file 4 **Supplemental Movie 3**. *nca(lf) *animal Click here for file

Additional file 5 **Supplemental Movie 4**. *nca(lf); unc-7 (e5) *animal Click here for file

Additional file 6 **Supplemental Movie 5**. *nca(lf); unc-7 (e5) *animal expressing UNC-7 by a pan-neuronal promoter Click here for file

Additional file 7 **Supplemental Movie 6**. *unc-7 (e5) *animal expressing UNC-7 by a pan-neuronal promoter Click here for file

Additional file 8 **Supplemental Movie 7**. *unc-7 (e5) *animal expressing Cys-less UNC-7 by a pan-neuronal promoter Click here for file

Additional file 9 **Supplemental Movie 8**. *unc-7 (e5) *animal expressing C191A UNC-7 by a pan-neuronal promoter Click here for file

Additional file 10 **Supplemental Movie 9**. *nca(lf); unc-7 (e5) *animal expressing Cys-less UNC-7 by a pan-neuronal promoter Click here for file

Additional file 11 **Supplemental Movie 10**. *nca(lf); unc-7 (e5) *animal expressing C191A UNC-7 by a pan-neuronal promoter Click here for file
