# Supplementary material for: A Co-operative Regulation of Neuronal Excitability by UNC-7 Innexin and NCA/NALCN Leak Channel
Source: Mol Brain. 2011 Apr 13;4:16. doi: 10.1186/1756-6606-4-16 (PMC3102621; doi:10.1186/1756-6606-4-16)
Supplement: Additional file 3 — Supplemental tables. [file 1756-6606-4-16-S3.DOC]

Additional file 3: Supplemental tables

**Table S1:** *unc-7* mutants do not display obvious synaptic transmission defects at NMJs

|  | | *unc-7* | | *unc-7* | |
| --- | --- | --- | --- | --- | --- |
|  |  | wild type | *unc-7(e5)* | wild type | *unc-7(hp121)* |
| mPSCs | N | 9 | 10 | 12 | 10 |
|  | Amplitude (pA) | 24.12±1.99 | 25.27±1.62 | 36.90±2.77 | 31.59±2.46 |
|  | Frequency (Hz) | 59.80±3.88 | 61.82±5.42 | 53.18±5.33 | 43.53±5.54 |
| Electric-EPSCs | N | 9 | 10 | 8 | 5 |
|  | Amplitude (nA) | 1.47±0.13 | 1.48±0.09 | 1.62±0.20 | 1.30±0.29 |
| ACh-EPSCs | N | 8 | 9 |  | |
|  | Amplitude (pA) | 975±122 | 906±72 |  |  |
| GABA-EPSCs | N | 7 | 10 |  |  |
|  | Amplitude (pA) | 1051±93 | 1014±64 |  |  |
| ACh photo-EPSCs | N | 8 | 8 |  |  |
|  | Initial Amplitude (nA) | 1.2±0.11 | 1.26±0.09 |  |  |
| GABA photo-EPSCs | N | 12 | 17 |  |  |
|  | Initial Amplitude (pA) | 668.2±66.5 | 693.7±72.1 |  |  |

**Table S2:** The functional interaction between the NCA and UNC-7 channels involves non-junctional UNC-7.

|  | | N | mPSC frequency (Hz) | mPSC amplitude (pA) | N | EPSC amplitude (nA) |
| --- | --- | --- | --- | --- | --- | --- |
| wild type | | 15 | 61.9±4.0 | 23.6±1.2 | 18 | 1.79±0.13 |
| *nca(lf)* | | 12 | 34.1±6.5 | 25.0±3.0 | 8 | 0.63±0.14 |
| *nca(lf); unc-7* | + *pan-neuronal UNC-7* | 8 | 36.2±9.9 | 25.9±5.2 | 7 | 0.52±0.15 |
|  | - *pan-neuronal UNC-7* | 4 | 25.3±4.6 | 17.6±1.8 | 4 | 1.47±0.17 |
| *nca(lf); unc-7* | + *Cys-less UNC-7* | 14 | 37.6±5.6 | 26.1±2.0 | 12 | 0.88±0.10 |
|  | - *Cys-less UNC-7* | 6 | 28.3±9.2 | 24.5±2.5 | 6 | 1.60±0.06 |
| *nca(lf); unc-7* | + *C191A UNC-7* | 10 | 45.4±7.5 | 22.3±1.5 | 10 | 1.20±0.12 |
|  | - *C191A UNC-7* | 7 | 28.4±7.5 | 22.6±1.3 | 4 | 2.05+0.12 |
